# Towards Integrated Management of Dengue in Mumbai

**DOI:** 10.3390/v13122436

**Published:** 2021-12-04

**Authors:** Prasad N. Paradkar, Pallavi R. Sahasrabudhe, Mrunal Ghag Sawant, Sandeepan Mukherjee, Kim R. Blasdell

**Affiliations:** 1 CSIRO Health & Biosecurity, Australian Centre for Disease Preparedness, 5 Portarlington Road, Geelong 3220, Australia; Kim.Blasdell@csiro.au; 2 Global Health Solutions, A.W. Vartak Marg, Vile Parle, Mumbai 400057, India; sahasrabudhe.p@gmail.com; 3 Department of Zoonosis, Haffkine Institute for Training Research and Testing, Parel, Mumbai 400012, India; mrunal@haffkineinstitute.org; 4 Department of Virology, Haffkine Institute for Training Research and Testing, Parel, Mumbai 400012, India; sandeepan@haffkineinstitute.org

**Keywords:** dengue, mosquito, India, Mumbai, novel strategies, Wolbachia, disease surveillance

## Abstract

With increasing urbanisation, the dengue disease burden is on the rise in India, especially in large cities such as Mumbai. Current dengue surveillance in Mumbai includes municipal corporation carrying out specific activities to reduce mosquito breeding sites and the use of insecticides to suppress the adult mosquito populations. Clinical cases remain either underreported or misreported due to the restriction to government clinics, missing the large private health care sector. There is a need for an integrated approach to manage dengue outbreaks in Mumbai. There are various novel strategies available for use that can be utilised to improve disease detection, mosquito surveillance, and control of mosquito-borne diseases. These novel technologies are discussed in this manuscript. Given the complex ecosystem of mosquito-borne diseases in Mumbai, integrating data obtained from these technologies would support the ongoing mosquito control measures in Mumbai.

## 1. Introduction

Diseases transmitted by vectors such as mosquitoes, are increasing in their geographic distribution as well as incidences worldwide [1]. More than 100 human diseases are caused by arboviruses (ARthropod-BOrne viruses), with vector-borne disease becoming an international medical and veterinary health concern [2]. Other than malaria, which leads to about 400,000 deaths worldwide every year [3], mosquito-borne viruses such as dengue, chikungunya, Japanese encephalitis, and Zika pose a huge burden on public health in tropical and subtropical regions. It is estimated that more than half of the world’s population is at risk of infection with these viruses [4].

Dengue is an acute viral infection in humans transmitted by *Aedes* spp. of mosquitoes. It is widespread throughout the world with a high prevalence around tropical and sub-tropical areas. More than 100 countries have demonstrated endemicity of dengue, including in Southeast Asia, Central and South America, Africa, the Western Pacific, and eastern Mediterranean regions [5]. The frequency and magnitude of epidemic dengue have increased dramatically in the past 50 years as the viruses and their mosquito vectors have both expanded geographically in the tropical and sub-tropical regions of the world [6]. Although the actual number of dengue cases remain underreported and many cases are usually misclassified, by one estimate these viruses cause more than 390 million infections per year, with approximately 20,000 deaths [7]. Dengue transmission typically shows periodicity with large outbreaks every 3–5 years in one region [8,9], often due to the temporal alternation of dominant serotypes [10]. The outbreaks also show seasonality with higher transmission correlated with increased rainfall, temperature, and mosquito population [11]. 

Dengue virus (DENV), the etiological agent, belongs to the family *Flaviviridae* and is divided into four distinct serotypes, DENV-1 to DENV-4, with 30–35% amino acid diversity [12]. Within each serotype, there are several genotypes that vary by approximately 3% at the amino acid level and typically do not exceed 6% nucleotide divergence. The genome of DENV is an enveloped positive and single-stranded RNA, approximately 11 kb in length, encoding a single polyprotein. The polyprotein is proteolyzed to form 10 proteins, which can be divided into two groups—structural and non-structural proteins. The three structural proteins consist of Capsid (C), Membrane (M), and Envelope (E); while the seven non-structural (NS) proteins consist of NS1, NS2A, NS2B, NS3, NS4A, NS4B, and NS5 [13]. 

Before World War II, most regions had only one or a maximum of two dengue serotypes circulating at any one time. During and after the war, initially due to the movement of troops and later due to increased travel/trade, many countries in Asia became hyperendemic; with co-circulation of all four dengue serotypes [6]. This increased the health burden of dengue, since co-circulation of multiple serotypes leads to antibody-dependent enhancement (ADE) which impacts disease severity as a result of partial host immune cross-protection [14].

The clinical presentation of dengue infection varies widely; with approximately 80% of infections remaining asymptomatic. The disease can manifest itself as a flu-like disease with sudden onset of fever, arthralgia, myalgia, retro-orbital headaches, maculopapular rash, leukopenia, and vascular leakage [15]. Approximately 1 in 20 patients with secondary dengue infection progress to develop a life-threatening disease called severe dengue, which includes dengue haemorrhagic fever and sometimes death due to shock. The inability to predict the severity of the dengue infection puts a significant burden on public health [16], sometimes with needless hospitalisations and huge economic losses through missed employment. Asymptomatic infections are increasingly recognised to play an important role in DENV epidemics and have been shown to contribute to onward DENV transmission [17]. Epidemiological studies, as well as laboratory studies using animal models, suggest severe disease outcomes are due to the phenomenon of ADE. Dengue infection by one serotype confers lifelong protection against that one serotype and brief (1–3 years) heterotypic protection [18,19]. However, when the person is exposed to a different serotype, the existing antibodies do not provide a neutralising effect. These heterotypic antibodies enhance the uptake of the virus by macrophages via the Fcγ receptor, enhancing the infection and in turn causing severe disease [14,20,21]. The presence of non-neutralising maternal antibodies is considered to be responsible for severe and often fatal dengue disease in children under the age of 5 years. Hence the circulation of multiple serotypes in a geographic region increases the likelihood of people being affected by severe disease [22,23]. 

Currently, there are no approved therapeutics against dengue disease, with patients usually treated symptomatically. The only approved vaccine for dengue, *Dengvaxia*, is not effective against all four serotypes and is only approved for use in children (9–16 years old) in endemic areas and who have had a laboratory-confirmed previous infection [24]. Although somewhat efficacious, the vaccine has been marred in controversy, with some reports showing vaccine leading to more severe disease in children [25]. There are other vaccine candidates still in development [26]. 

Vector control with mosquito population suppression remains the most effective weapon against dengue outbreaks. Vector density and distribution is strongly influenced by environmental factors and with increasing temperature, there is increased geographic spread of mosquitoes and the disease [27,28]. The virus is transmitted by *Aedes* spp. of mosquitoes and the use of insecticides to reduce the population is traditionally used by public health agencies worldwide. More recently, mosquitoes have been shown to become resistant to traditional insecticides [29,30,31,32], highlighting the need for innovative approaches. Other than traditional vector control methods, there are various next-generation approaches under consideration or in use to reduce the impact of dengue and other mosquito-borne diseases. The strategies range from targeting the aquatic stage of mosquitoes using larvae-eating fish [33], to use of fungus targeting mosquitoes [34,35], to use of the endosymbiotic bacteria, *Wolbachia*, to reduce the competence of mosquitoes to transmit the virus [36]. More recently, genetic approaches to either reduce population [37,38] or to reduce vector competence [39,40,41], have also been tested in a limited capacity in the field. Although long-term impacts of these approaches are being established, these novel strategies may provide a way forward for tackling these diseases.

## 2. Dengue Burden in India

While Southeast Asia contributes to 53% of the global dengue disease burden, India alone accounted for 34% of the apparent dengue infections worldwide in 2010 [42,43]. Although dengue is a notifiable disease in India, some estimates suggest that the disease is underreported [44,45], so actual numbers are likely higher. Dengue transmission follows a seasonal and cyclical pattern, coinciding with rainfall [46], mosquito population densities and population immunity status [47]. Over the last 20 years there has been a significant increase in the incidence of dengue in India, especially in urban areas. This has been due to increased urbanisation and the resulting ‘Urban Heat Island effect’ seen in intensely populated urban areas [48,49], as well as more travel and trade [46]. Increased urbanisation usually includes poor and sometimes temporary housing, the absence of piped water supply and insufficient waste management, providing ideal mosquito breeding conditions [6,50]. The *Aedes aegypti* mosquito, which is responsible for dengue transmission, is highly adapted to this urban environment.

India’s National Vector Borne Disease Control Programme (NVBDCP) reported 157,315 dengue cases in 2019 with 166 deaths, with most of the cases in the Western states, such as Gujarat, Maharashtra, and Karnataka [51]. These figures, however, consist of data only from government hospitals, neglecting India’s large private health sector, which covers approximately 80% of all dengue-related healthcare visits [52,53]. One estimate suggests the real case number to be 200–300 times more than the reported cases per year [54]. There is a need for an enhanced health surveillance system, including both public and private healthcare, with better reporting, early detection, and the capture of associated spatial information, for better management of the disease outbreaks and future prediction. India’s first reported dengue epidemic was in 1963–1964 when dengue gradually spread from the country’s eastern regions to its northern states and progressively to the whole country by 1968 [55]. The epidemiology of dengue virus and its prevalent serotypes has been ever-changing. Although before the epidemic, there was only the circulation of DENV2 serotype, but by the late 60s–early 70s all four serotypes were circulating [56]. There have been recurring outbreaks of dengue in India in the following decades, mostly with a single serotype circulating at any given time. However, since the late 90s, India has seen multiple serotypes circulating simultaneously, leading to increased incidences of severe dengue disease [57]. 

## 3. Dengue Surveillance in India

A passive surveillance program for dengue was set up by the Indian government after a large scale outbreak in 1996, where 16,517 cases and 545 deaths were reported [43,51]. Passive surveillance relies on disease notification by healthcare professionals who are required to report all suspected cases of reportable diseases. However, this does not include the private health care sector, reducing its sensitivity for early detection of an outbreak. Local authorities often admit that the reported case numbers could be a gross underestimation of actual incidence with only about 0.35% of clinically diagnosed cases reported, providing a misleading picture of the actual dengue burden [54]. Official disease surveillance systems come under the responsibility of state/local government, although the data is centralised by the NVBDCP. NVBDCP is the government’s central nodal agency for the prevention and control of vector-borne diseases. It is responsible for reporting on suspected and confirmed dengue cases, facilitating prevention activities, health awareness, and ensuring immediate outbreak response. The NVBDCP has developed guidelines for the prevention and control of dengue, assisting state governments and local authorities with their programs [51]. The Integrated Disease Surveillance Programme (IDSP), operating under the National Centre for Disease Control and Director General of Health Services of the Ministry of Health and Family Welfare, aims to improve India’s regional and national disease surveillance and response [52]. Its key objectives are enabling surveillance in epidemic-prone diseases, monitoring disease trends, facilitating the timely response to outbreaks, as well as integrating the local data with state and national database. Local authorities, such as municipal corporations, are responsible for the effective implementation of vector control and disease monitoring programmes.

*Ae. aegypti* and *Ae. albopictus*, both common vectors of the dengue virus, are present in India [58,59,60]. *Ae. Aegypti* is well adapted to the urban environment, while *Ae. albopictus* is suited to peri-urban areas. With increasing urbanisation, leading to increased construction, poor water management, water tanks and heavily populated areas with substandard housing and squalor, *Ae. aegypti* is slowly displacing *Ae. Albopictus* [61]. Vector control using insecticides is a commonly used method in India for the prevention of vector-borne diseases. There are several reports which have demonstrated resistance of mosquito vectors to anti-larval substances like DDT and dieldrin but retained susceptibility to malathion [62,63]. Peridomestic thermal fogging, common in many regions especially during the monsoon season, reduces the resting and biting of mosquitoes for several days [64]. Other vector control measures include environmental management with detection and elimination of mosquito breeding sources, management of stored water, use of chemicals in breeding containers as well as public education around the use of mosquito repellents and bed nets [51]. Community participation in the identification of mosquito breeding spots in a neighbourhood is an essential part of reducing the mosquito population and is also utilised in some areas.

## 4. Dengue in Mumbai

Mumbai, located in the Western state of Maharashtra, is the most populous metropolitan city in India, with an estimated population of more than 20 million people. A large part of the population lives in semi-permanent housing and squalor, with poor water supply. This leads to people storing water in large containers, either inside or outside their housing, inadvertently providing habitats for the breeding of mosquitoes. Mumbai receives an average rainfall of 2386 mm during the monsoon months of June to September, during which time there is an increase in mosquito numbers leading to increased dengue cases [65].

In Mumbai, laboratories conducting RT-PCR (Reverse Transcription-Polymerase Chain Reaction) for early detection of dengue are predominantly in the private sector, with associated high cost for testing, making it unaffordable for government and municipal hospitals [65]. The Molecular Diagnostic Reference Laboratory at an infectious disease hospital in Mumbai, is the public health laboratory of the Municipal Corporation of Greater Mumbai (MCGM) and receives samples from all tertiary and secondary health care facilities under MCGM for dengue RT-PCR in the early phase of illness (0–7 days). The detection and response of dengue outbreaks remain with the MCGM, which collects the data from the government hospitals and responds mainly via vector control strategies in that municipal ward. It also runs community awareness campaigns during the season to reduce the spread of the outbreak. Despite this, Mumbai experiences dengue outbreaks every year, with varying intensity [51]. Several factors have led to the increased frequency and duration of dengue outbreaks seen in recent years. These include high human population density which provides susceptible hosts for mosquitoes, increased construction sites, squalid living conditions, and lack of waste removal which along with high rainfall during the monsoon lead to water logging [65]. There is also increased migration into the city for employment and the daily movement of people for work activities increases the risk of disease introduction and spread.

MCGM undertakes specific activities to prevent outbreaks of mosquito-borne diseases. These include inspection of water bodies, such as wells, water tanks, and swimming pools. MCGM also carries out removal of breeding sites such as water pipes, tyres, and household plants, which are all known to host mosquito larvae. These activities are performed in preparation for the monsoon arrival in June. In recent years, MCGM has also used aerial drones to identify possible breeding sites inside congested areas and to spray insecticide to kill mosquito larvae. One of the major actions taken by MCGM is vector control by peridomestic thermal fogging and the use of insecticides to suppress the adult mosquito population and biting. This is also increased in response to reporting by the general public of increased mosquito bites or detection of dengue cases. During the monsoon months, MCGM performs passive surveillance based on dengue case reporting [66]. Although there is some awareness in the community and by policymakers regarding mosquito population and dengue outbreak risk, compliance is a major factor [67].

With the complexities of a modern megapolis, Mumbai boasts to be the financial capital of India. With that, however, comes a need for appropriate planning of health systems for early detection, suitable preventative measures, and control of any outbreak with a targeted response. During 2020, several cities and countries around the world experienced large outbreaks of dengue; possibly exacerbated by COVID-19 related lockdowns. Singapore reported 34,844 cases in 2020 [68], much more than in previous years. Indonesia has recorded more than 95,000 infections [69], while Thailand reporting more than 71,000 cases in 2020 [70]. Sri Lanka recorded more than 31,000 cases during 2020 [71]. With these record increases of dengue cases in recent years, a large city such as Mumbai may also experience a large dengue outbreak of its own in coming years.

For better management of dengue outbreaks, there is a need for an integrated response. This should include surveillance for dengue disease, active and passive vector surveillance activities, management of dengue cases, early identification of hotspots for transmission, targeted response to reduce transmission and community engagement to maintain low dengue case numbers. This requires the coordination between various public and private stakeholders such as health professionals, field entomologists, diagnostic laboratories, private health insurance companies as well as local governments (municipalities), community leaders, and data scientists. There is also a need for continuous feedback and evaluation of these interactions and resulting public health measures for improvements.

**Figure 1:**
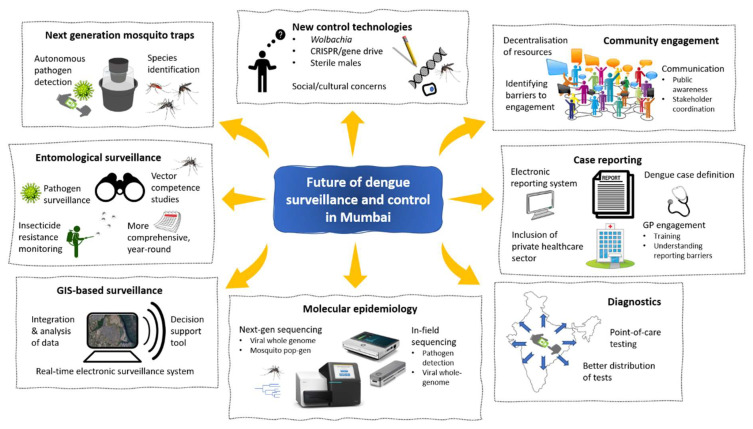
Integration of novel technologies in dengue surveillance and control. Novel technologies used in combination with traditional strategies can significantly reduce impact of mosquito-borne disease in large cities such as Mumbai. Improving clinical case reporting to integrating mosquito surveillance in a digital system will detect disease outbreaks early. Using Wolbachia-based mosquito control strategies can reduce incidences of dengue significantly.

Although IDSP, NVBDCP, and MCGM all operate within Mumbai and work in parallel at different scales, there is a need for an integrated approach for monitoring cases for surveillance and appropriate targeted response (Figure 1). An enhanced public health surveillance system, based on accurate and early data, is needed for the prediction and detection of dengue outbreaks and ultimately for effective outbreak response.

## 5. Future of Dengue Surveillance and Control in Mumbai

### 5.1. Improvement in Dengue Case Reporting

One of the major first steps of outbreak response is accurate reporting of dengue cases. It has been estimated that most of the dengue infections remain unreported or misreported [72,73]. This is mainly because reporting is limited to government clinics and diagnostic labs that completely excludes the private healthcare sector, only permitting their optional reporting [54]. Although dengue is a notifiable disease to the state government, full implementation of the reporting by healthcare professionals, especially GPs, has been a problem historically [45]. The issues and barriers related to reporting are not well understood. They include imprecisions of WHO case definition relevant to India, which helps classification and case management rather than reporting [45]. For this, the clinician needs to be well trained to suspect dengue and report accordingly. There are possibly several other impediments to reporting cases, such as community stigma, lack of incentive to health professionals, complicated reporting procedures and lack of feedback from the public health authority [45]. There needs to be a systematic study of the impediments to dengue reporting in Mumbai and the identification of possible incentives to increase reporting. There needs to be engagement between community and health professionals, as well as provision of an easy electronic reporting system, with accompanying information like geographic data and patient clinical data. This data will help the public health authorities to assess incidence/hospitalisation rates as well as target the response to the outbreak. Accurate reporting of dengue case numbers will also provide an accurate picture of the disease burden on public health in Mumbai.

### 5.2. Supporting Dengue Diagnostics

Diagnosis of dengue infection is routinely done by IgM capture or by NS-1 antigen using ELISA kits on patient serum depending on the day of illness (assay developed by National Institute of Virology, Pune). The ELISA kits are provided by the central government to each state as per the state’s demand, which is typically based on the number of cases in the previous year, leading to over or underestimation of actual cases [73]. ELISA IgM is present in the sera of patients 5 days post infection in acute primary infection, but the IgM response may be low or sometimes even absent in secondary dengue infection. Other laboratory tests for severe dengue disease include blood with atypical lymphocytosis and thrombocytopenia (<65,000 per uL). Molecular methods (reverse transcriptase PCR), which are more sensitive and can be used early in infection, are being increasingly used and can differentiate dengue serotypes. The use of good dengue diagnostic tools is critical for laboratory confirmation of severe disease, counting the case fatality rates, identifying circulating strains, and calculating the total incidence rates. Studies suggest that antigen detection and RT-PCR are the most sensitive tests during the early period of illness whereas, beyond the third day, IgM antibody detection was found to be the most sensitive method of dengue diagnosis [74,75]. However due to cross-reactivity with other circulating viruses, such as Zika virus, interpreting results can be difficult and can lead to misdiagnosis [76]. Alongside this passive surveillance, there is also a need for active and sentinel surveillance to detect circulating dengue. This may be achieved through the detection of dengue using rapid test kits for IgM capture in a population. There are some rapid diagnostic tests (RDTs) available on the market, however, they lack independent verification and show variable sensitivity and specificity [77]. There is a need for a standardised, rapid, cheap, and scalable point of care surveillance kits, which can be used during an outbreak. There are currently various platforms available that employ novel technologies such as lateral flow using antigen, CRISPR-based detection, and nanobody-based detection. This rapid test could act as a surveillance tool and combined with easy reporting, would enable a quick public health response. These surveillance tests could then be followed up with standard laboratory tests, such as platelets and other markers, for clinical management of the patient.

### 5.3. Improved Entomological Surveillance

Parallel to the surveillance of dengue cases, entomological studies of mosquito vectors should be regularly conducted, both in urban and semi-urban areas [78]. Known dengue vectors, *Ae*. *aegypti*, *Ae. albopictus,* and *Ae. vittatus* are present in the Mumbai region [79,80]. Although these are the major vectors for dengue, there may be additional unknown vectors present that may play a role in transmission or serve as reservoirs. Mumbai, with its semiurban areas and mangroves is also home to several other species of mosquitoes with unknown vector competence [81]. MCGM conducts regular surveillance of mosquito abundance at various sites throughout the year, focusing on the pre-monsoon season, in line with the NVBDCP guidelines [66]. This involves using mosquito traps as well as identifying mosquito breeding sites. Mosquito collection from fixed or random sites is used for density studies, which determine the population control locations. Molecular testing for rapid detection of species and pathogens from collected mosquitoes, which could improve both outbreak prediction and response, is not undertaken routinely. There is also a need to perform year-round surveillance to determine mosquito population dynamics, to understand the seasonal changes as well as identifying refuge spaces harbouring mosquitoes during the winter months. MCGM also undertakes routine treatment of sites with insecticides (including larvicides). However, this could be more effective with an improved understanding of the emergence of insecticide resistance in mosquitoes. A previous large-scale study that assessed the susceptibility of *Ae*. *aegypti* to insecticides found that mosquitoes from various locations were still susceptible to temephos, fenthion, and malathion, whereas a low level of DDT resistance was noticed in field-collected *Ae. Aegypti* [62]. However, in a more recent study, *Ae. aegypti* found primarily breeding in fire buckets in the Mumbai port areas, were found to have high levels of insecticide resistance to temephos and fenthion [82]. There is a need to continuously monitor the insecticide resistance of *Aedes* mosquitoes in Mumbai for effective control.

### 5.4. Use of Novel Mosquito Traps for Surveillance

Traditional mosquito traps, such as gravid [83,84] or light traps [85,86], have been used for surveillance but are also considered as tools for mosquito control if they are sufficiently specific in attracting target species. These traps are placed across a landscape and monitored for mosquitoes at definite intervals, usually weekly. The mosquitoes are processed in the laboratory for species identification by morphology or molecular testing. Detection of pathogens in collected mosquitoes is also carried out using molecular methods. In recently trialled Passive Box Traps, viral nucleic acids are deposited by the mosquitoes while sitting on a (honey-baited) filter paper card. The card can then be removed and subjected to molecular analyses [87]. These may thus be suitable for the collection of mosquitoes for pathogen surveillance. Testing individually caught mosquitoes offers a very precise means of determining the infection rate. However, mosquito populations often have very low carriage rates and, to increase the probability of detection, large numbers of mosquitoes are usually required. Researchers have recommended an intensified sampling strategy at sites where potential vector mosquitoes are abundant, or in areas with a history of arbovirus circulation [88]. Such an approach would be more cost-effective and increases the probability of detection which is important for early outbreak detection. Next-generation traps are also being developed to improve the collection, species detection, and autonomous pathogen identification [89]. In Mumbai, MCGM uses mosquito traps at various sites based on historical data. However, data collection could be improved if used in combination with mathematical modelling and new technologies to understand mosquito species, seasonal variability in populations, and changes due to various factors such as urbanisation and land use. The use of next generation sequencing (NGS) in determining mosquito population structure is important in understanding the movement of mosquitoes at different spatial scales [90]. Combined with metabarcoding, which is barcoding of mosquito DNA to allow for simultaneous sequencing and taxonomic identification, it can allow the rapid identification of large numbers of mosquito species with simultaneous screening for pathogens [91].

### 5.5. Molecular Epidemiology to Support Surveillance

Molecular testing by RT-PCR has become the most used tool in diagnostic and research laboratories for detecting infection in mosquitoes [92] or human samples [93]. With advances in sequencing technologies, it is possible to sequence the whole genome of the virus in laboratory or field settings. In recent times, NGS platforms have moved from research studies to routine use in the clinical microbiology laboratory [94,95], especially in well-resourced settings. For clinical laboratory settings, there is also a trend towards smaller and less expensive bench-top sequencing instruments such as Ion Torrent or Illumina MiSeq system, compared with larger systems requiring huge infrastructure costs. With newer technologies such as MinION, a portable sequencing platform by Oxford Nanopore Technologies, it is possible to directly sequence from a clinical or environmental sample in field conditions [96]. However, this technology has its challenges with a requirement of high DNA or RNA input and high error rates [97]. The availability of the whole genome provides an additional dataset and can be used to inform not just pathogen diagnostics but also provides valuable information about the epidemiology of the outbreak. Laboratory testing should include, wherever possible, sequencing of dengue isolates from patient samples. Comparing DENV genome sequences to those already in existing database may be useful in determining changing epidemiology and predicting the severity of the outbreak. This would not only support diagnosis but would also allow identification of the serotype(s) and genotype(s) forming a part of molecular epidemiology predicting and determining the outbreak spread [98]. Although these capabilities exist in India, their use in public health settings remains limited, mainly due to the requirement of large investments in lab capacity and bioinformatics.

### 5.6. Use of Geographic Information System (GIS)-Based Surveillance

GIS-based technologies, such as DengueNet [99] or DengueMap [100], have been used to monitor dengue disease transmission internationally. However, there is a need for a real-time electronic surveillance system with spatial information more locally in Mumbai, which will aid in generating a platform for capturing, integrating, and analysing disease-relevant information for use by the local public health system. This platform would not only serve as a database for disease incidence across a region but could also be used as a decision support tool for identifying transmission risk at a fine spatial scale. By utilising epidemiological as well as entomological data, site-specific response could be triggered by public health officials, focusing the resources such as insecticide uses, community campaigns, and active surveillance. This platform has been used in Mexico for dengue surveillance and it made a significant impact on mosquito and disease control activities [101]. Similar platforms can be developed for use by public health authorities in Mumbai to focus strategies for controlling mosquito population and reduce dengue impact.

### 5.7. Improved Community Engagement and Response

Strong and long-term political and social backing is a critical component for the sustainability of these intervention strategies. Community-based programs that enable local authorities to participate in eliminating mosquito breeding sites are cost-effective in ensuring dengue control in any region [102,103]. Community mobilisation, however, requires decentralisation of resources and a high level of coordination among all stakeholders, including clinical health workers (public and private), public health officials, community leaders and legislators [104]. Lack of coordination between health agencies and communities may lead to an ineffective response. Mumbai municipal corporation undertakes several such information drives during pre-monsoon months. This has led to a medium-high level of awareness in people and communities, although compliance remains an issue due to various factors. Understanding these barriers to compliance by engaging with communities would have a massive impact on reducing the mosquito population and in turn, dengue incidence.

### 5.8. Use of Novel Technologies for Mosquito Control and Transmission Prevention

Alongside the use of traditional methods to reduce mosquito numbers, advances have been made to develop novel mosquito control strategies. The use of Wolbachia to either reduce dengue transmission has been studied for the last 20 years [36] and has been implemented in several countries. A recent study showed Wolbachia deployment in Yogyakarta (Indonesia) reduced dengue incidence by 77% and dengue hospitalisations by 86% [105]. Another approach is use of Wolbachia to reduce mosquito populations by releasing Wolbachia infected male mosquitoes, which produce non-viable offspring. A recent study conducted at Innisfail (Australia) showed that release of Wolbachia infected *Ae*. *aegypti* males significantly suppressed the mosquito population [106]. These Wolbachia-based approaches are not mutually exclusive and can be implemented taking local conditions into account. With complex geographical, social, and ecological conditions in Mumbai, after appropriate risk analysis, these novel approaches can reduce the impact of dengue in a sustainable manner. This would require early engagement with local stakeholders including communities and regulatory bodies.

More recently, there have been significant advances in genome engineering, which has opened the door for the use of modified mosquitoes to either reduce population [37,38,39,107] or to reduce the competence of mosquitoes to transmit diseases [40,41]. Biotech firm Oxitec recently launched a field test of its genetically modified mosquitoes in the United States, with results to be presented to the US Environmental Protection Agency [108]. Oxitec has previously field-tested the mosquitoes in Brazil [109], Panama, the Cayman Islands, and Malaysia [110]. Despite the exciting possibilities, there remain some important challenges to their widespread introduction, such as regulatory considerations, logistical difficulties, and technical issues, as well as social and cultural concerns, which can influence the acceptance of these methods. Due to the absence of national and international guidelines, recently multidisciplinary researchers around the world outlined their commitments to conducting field studies using genome engineering technologies responsibly [111].

For these novel methods to be successfully utilised in regions such as Mumbai, there needs to be robust local entomological capacities and capabilities with background studies to determine the local mosquito population and gene flows to understand mosquito movements. The development of insecticide resistance should also be tracked regularly to determine the viability of using these measures. A recent modelling study, investigating the success or failure of Wolbachia-based approaches, showed the importance of matching insecticide resistance levels in release stocks to those in the target natural populations during Wolbachia deployment [112]. These baseline data will aid in putting appropriate control measures in place, as well as helping to determine the success of such measures over time.

## 6. Conclusions

With growing human and mosquito populations and increasing incidence of dengue in large cities such as Mumbai, there is a need for an integrated response to reduce the impact of dengue on the local vulnerable population. Although India’s economic developments have been enormous, large disparities exist in health and social disciplines. The surveillance system relies on public reporting units for clinical as well as entomological data. Organisationally, the National Vector Borne Disease Control Program (NVBDCP) is responsible for vector and disease control, while the Integrated Disease Surveillance Programme (IDSP) is responsible for reporting and data integration. In Mumbai, the local municipal organisation is responsible for vector control and epidemiological reporting. There are various barriers to accurate reporting of dengue data include not involving private healthcare, complicated diagnostic procedures, and system fragmentation. Currently, mosquito surveillance in Mumbai is passive and mainly focused on larval surveys. There is also a need for baseline studies to understand the mosquito population and seasonal changes, for appropriate planning of control measures. Developing surveillance capabilities is essential for the implementation of strategies to reduce dengue outbreaks, whether these be traditional or novel approaches. Finally, integration of epidemiological and entomological data, along with relevant stakeholder engagement including communities, is important in developing a decision support system. This system on a digital platform could serve as an early warning system for dengue outbreaks, enabling a faster and more effective response. Increasing our understanding of mosquito population dynamics and disease epidemiology in Mumbai, along with the use of novel technologies such as a decision support system and Wolbachia to control infection transmission, would support traditional dengue surveillance and mosquito control measures in Mumbai.
